# Assessment of Turning Polytetrafluoroethylene External Cylindrical Groove with Curvilinear Profile Tool

**DOI:** 10.3390/ma16010372

**Published:** 2022-12-30

**Authors:** Jing Ni, Bokai Lou, Zhi Cui, Lihua He, Zefei Zhu

**Affiliations:** School of Mechanical Engineering, Hangzhou Dianzi University, Hangzhou 310018, China

**Keywords:** polytetrafluoroethylene, grooving, tool contact, surface roughness

## Abstract

Polytetrafluoroethylene (PTFE) is extensively used in equipment used for manufacturing semiconductor components and wet etching equipment. However, achieving ideal dimensional accuracy when cutting PTFE is challenging. In this study, we performed cutting experiments using a curvilinear tool and analyzed cutting force, cutting temperature, groove width, and surface roughness in PTFE grooving. The results indicated that the cutting force was most notably affected by the feed rate in Stage I of grooving. The rate of change in cutting force was the largest in Stage II because of the increase in the tool contact area. In Stage III, the shear area of the rake face was the largest, and the cutting force tended to be stable. The groove width was measured with a minimum error rate of 0.95% at a feed rate of 0.05 mm/rev. Moreover, the groove exhibited a time—independent springback. The minimum groove surface roughness was 0.586 at a feed rate of 0.05 mm/rev. The ideal feed rate was 0.05 mm/rev with groove width, surface quality, and chip curl as the key parameters. The processing parameters obtained in this study can be applied to actual production for the optimization of manufacturing accuracy.

## 1. Introduction

Owing to its excellent stability and high-temperature thermal insulation, polytetrafluoroethylene (PTFE) is extensively used in semiconductor manufacturing equipment [1]. PTFE workpieces are typically molded through compression sintering instead of injection molding using conventional thermoplastics. However, the mechanical properties of PTFE hinder the cutting of semiconductor components, which have complex shapes and require high precision. The low stiffness and high elastic modulus of PTFE cause chip curling [2,3], and its high linear elastic coefficient causes thermal deformation. Therefore, PTFE cutting technology should be developed to optimize the manufacturing process and improve the surface quality of the parts.

Studies have reported the effects of cutting parameters on the surface quality of PTFE and other polymers. Cui et al. [4] conducted orthogonal cutting experiments on PTFE. When cutting at high cutting speed and depth, severe plastic deformation, such as tearing and ripping up, occurs during chip formation, leading to excessive cutting and irregular burrs on the workpiece surface. Na et al. [5] performed PTFE multifactor turning experiments to optimize the machining parameters of the traditional regression model based on experimental results using the multi-objective algorithm NSMTLBO. The optimal machining parameters obtained using the model can be used to achieve the optimal material removal rate and surface quality. Fetecau et al. [6] performed turning experiments on PTFE through analysis of variance (ANOVA) and established a model of cutting forces and surface roughness to process variables. Ni et al. designed a model of the cutting load in PTFE drilling [7] and concluded that the tool contact coefficient and friction coefficient are considerably affected by feed rate and that the cutting coefficient is considerably affected by spindle speed. Gan et al. [8] used liquid nitrogen to lower the processing temperature and thus suppress the generation of burrs and subsurface damage layers on the groove surface during PTFE milling. Petro et al. [9] conducted turning experiments on three polyether–ether–ketone (PEEK) polymers and demonstrated that surface roughness can be effectively improved by increasing the feed rate. A higher feed rate increases the periodicity of the profile; this phenomenon also occurs in fiber-reinforced composites. Ji et al. [10] investigated the difference between pure PEEK and fiber-reinforced PEEK in SPDT. The results indicated that the homogeneity of the material affects the fluctuations of the cutting force, and the roughness of the cutting fiber increases when a fracture occurs. Jogi et al. [11] performed a grey relational analysis, which revealed that the roughness of PEEK turning is most notably affected by cutting force. Goel et al. [12] used the Taguchi method to design PMMA turning experiments and summarized the influence of surface roughness on the feed rate. The designed grey grade values indicated that the tip radius is the prominent factor that influences the micro-waviness of PMMA. Trifunović et al. [13] conducted turning operations on POM-C and discovered a pattern by varying the cutting parameters against the material removal rate to measure the energy generated by the cut. The large sample analysis of the cutting experiment cannot only use the traditional regression analysis. Gupta et al. [14] used various artificial intelligence algorithms (e.g., SVR/GA-ANN) to optimize the turning process parameters; the results indicated that the artificial intelligence algorithms were more accurate for the large sample dataset generated during cutting. The effective cutting parameters were selected for practical machining to achieve optimum results in terms of machined surface roughness and tool wear. Chabbi et al. [15] used ANN methods for the optimization of cutting parameters and combined multi-objective optimization, such as quality optimization, productivity optimization, and quality and productivity optimization, using desirability functions.

Studies have established various cutting methods using geometric analysis tools. Ni et al. [16] used the finite element method to simulate shear band morphology at different cutting depths. The contact behavior and shear stress equation of a curved insert in the cutting process are different from those of conventional cutting tools. The cutting force of the insert is primarily affected by the thickness of the curved shear zone and the depth of cutting. Weng et al. [17,18] modified the Johnson–Cook model to calculate the shear stress by accounting for size effects and discretizing the cutting edge at the front of the tool. Size is a key factor that influences the cutting process of the curved insert. The analysis model was optimized by considering the edge effects of different feeds. Cutting precision and chip formation are affected by material extrusion and flow due to tool extrusion in grooves. Lu et al. [19] investigated the depth variations in the plowing V-shaped groove at different cutting depths through experiments and finite element methods. The results indicated a correlation between material flow and compressive stress during plowing. The influence of cutting parameters on chip shape and surface quality should be investigated. Li et al. [20] varied the tip arc length during turning experiments, and the thickness of the white layer on the machined surface increased with the increase in the tip arc length. During the cutting process, the properties of elastoplastic materials affect the roughness of the resulting surface. Xu et al. [21] discovered that the chip morphology obtained by using inserts of different shapes during grooving is not consistent; in particular, excellent chip morphology and machined surface quality are achieved using a grooving insert with a side wiper edge. Based on the theory of plastic transverse flow, models of tool circular feed rate and surface roughness were established. Vibration is inevitable in grooving, and several studies have reported the correlation between machining parameters and vibration. Hocheng et al. [22] analyzed the characteristics of roughness curves and established a surface roughness model of Rq by accounting for the influence of tool geometry and low—frequency vibrations. Tian et al. [23] performed finite element simulation and cutting experiments to investigate the effects of insert vibrations (including those along the cutting direction and the feed direction) on diamond—turning surface roughness.

Although various methods for cutting PTFE and polymer composites have been proposed, few studies have focused on the grooving of pure PTFE. PTFE bellows are used in ultra-clean flow control pumps, and the external cylindrical groove structure of the bellows determines the efficiency and service life of reciprocating motion [24]. Therefore, investigating the grooving process is crucial to improving the quality of external cylindrical grooves. In the present study, we performed single—factor experiments to analyze the different stages of grooving. Furthermore, we defined different contact zones to analyze the cutting force variations and chip morphology. Then, we measured the groove width of the workpiece and calculated the decrease rate under different parameters. Finally, the groove width was investigated and the machined surface topography was measured to determine the optimal parameters for cutting PTFE annular grooves.

## 2. Experiment

PTFE manufactured by VALQUA Plastics (Austria) was used as the material to be subjected to grooving. The diameter and length of the workpiece were 90 and 120 mm, respectively. Table 1 presents a summary of the mechanical properties of the workpiece.

**Figure 1:**
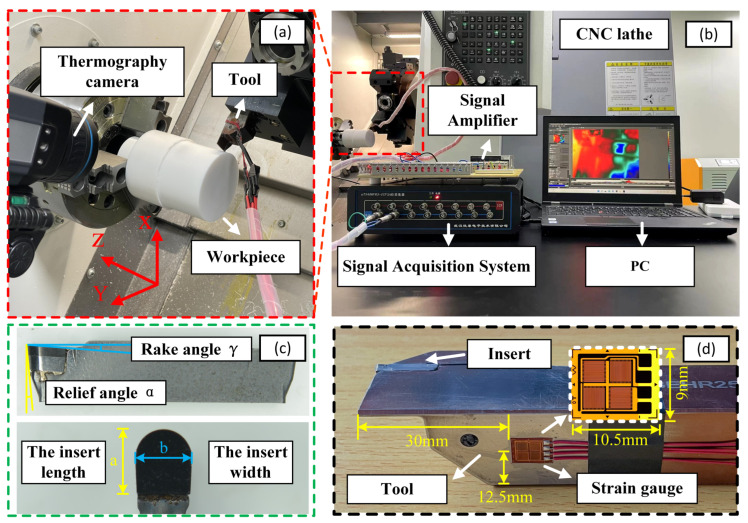
Experimental setup: (**a**) cutting area; (**b**) signal acquisition system; (**c**) parameter diagrams of the curvilinear profile tool; and (**d**) sensor location.

As illustrated in Figure 1, a Schiess VIVA TURN 2 CNC lathe was used to perform cylindrical dry grooving experiments. Using this experiment design, a groove width $D_{a}$ of 2 mm and a groove depth *H* of 18 mm were obtained. As recommended in the *Fluoropolymer Processing Handbook* [25], the following cutting parameters were used in the present study: the cutting speed was 400 rpm, and the feed rates were 0.05, 0.10, 0.15, 0.20, 0.25, and 0.30 mm/rev. Owing to its high hardness, wear resistance, and heat conductivity, polycrystalline diamond (PCD) was selected to improve processing efficiency. Therefore, a PCD insert was used for the grooving experiments in this study [26]. We used a curvilinear insert with a length *a* of 5 mm and an edge width *b* of 2 mm; the rake angle γ and relief angle α were 5° and 2°, respectively. The edge profile resembled a semicircular curve.

Cutting force signals were collected using two full—bridge strain gauges, and the signals in each channel were independently amplified and input to a computer in a UT3408FRS-TCP dynamic signal analysis system. The distance between the strain gauges and the tool tip was 30 mm, and 12.5 mm was the distance between the gauges and the tool side. The vertical and horizontal dimensions of the strain gauges were 9 mm and 10.5 mm, respectively. The specific experimental parameters were as follows: the sampling frequency was 128 kHz and the amplification factor was 4.95. The cutting temperature was measured using a FLIR A655c thermography camera. The cutting area between the grooves was set as the measurement area, the atmospheric emissivity was set at 0.95, and the measurements were taken from a distance of 2 m.The surface 3D topography was observed with a KEYENCE VW-9000 optical microscope.

## 3. Results and Discussion

### 3.1. Analysis of Cutting Force

**Figure 2:**
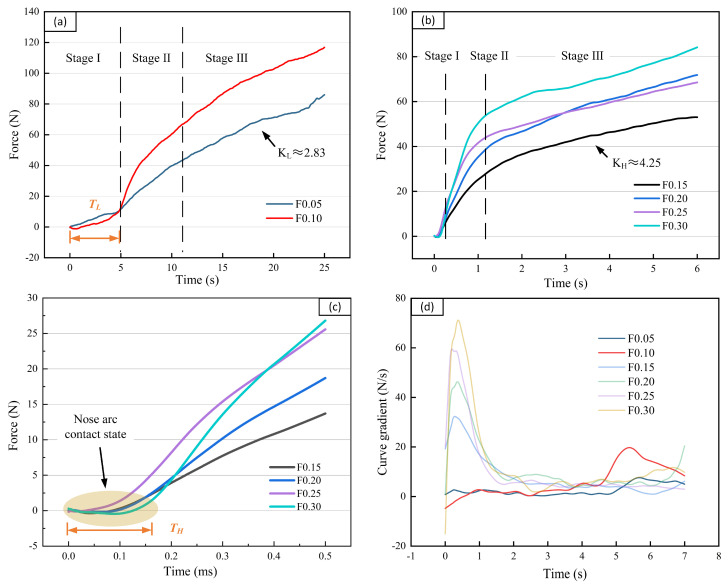
Comparison of cutting force curves at different feed rates: (**a**) cutting force at low feed; (**b**) cutting force at high feed; (**c**) enlarged curve of Stage I at high—feed rates; and (**d**) rate of curve at different feed rates.

The cutting force curve for each feed is displayed in Figure 2a,b. The results were divided into two feed conditions based on the difference in trend. Low—feed conditions were 0.05 and 0.10 mm/rev, whereas high-feed conditions were 0.15, 0.20, 0.25, and 0.30 mm/rev. Figure 2d shows the changes in the cutting force slope at different feed rates, and it can be seen intuitively that the change trend in the low-feed conditions is different from that of the high—feed conditions. The cutting force at different feeds can be divided into three parts according to the slope change. The cutting force curve plotted according to the experimental result was divided into three stages: Stage I, Stage II, and Stage III. Stage I was defined as the period when the slope was less than 5 at low feed and less than 16 at high feed. The duration of Stage I is $T_{L}$ at high feed and $T_{H}$ at low feed. The cutting force for both low feed and high feed rises rapidly, and this period is Stage II. When the trend of the curve slows down, the cutting force enters Stage III. The slope of this stage is $K_{L}$ at low feed and $K_{H}$ at high feed. Figure 2a displays the cutting force curve under low—feed conditions. The duration of Stage I was $T_{L}$, which was the stabilization time, and the cutting force stabilization time was 5 s. The cutting force began to increase in Stage II. In Stage III, the cutting force gradually increased and reached the maximum value. $K_{L}$ denotes the rate of change in cutting force in Stage III at a low feed rate. The results indicated that the same $K_{L}$ (=2.833) can be obtained at feed rates of 0.05 and 0.10 mm/rev.

As illustrated in Figure 2b, a different trend was observed for a high—feed cutting force. The high—feed cutting Stage I ended quickly, and Stage II was entered. As illustrated in Figure 2c, a flat Stage I was achieved under high—feed conditions by increasing the duration of Stage I. The duration of Stage I at high feed was denoted by $T_{H}$. When the feed rate was 0.30 mm/rev, Stage I was the shortest and $T_{H}$ was 0.08 s. In addition, the rate of change of cutting force was the highest in Stage II at a high feed rate. Stage III constituted most of the total cutting time. The rate of change in cutting force in Stage III was denoted by $K_{H}$. The value of $K_{H}$ for each parameter was constant (≈4.25) under high—feed conditions.

**Figure 3:**
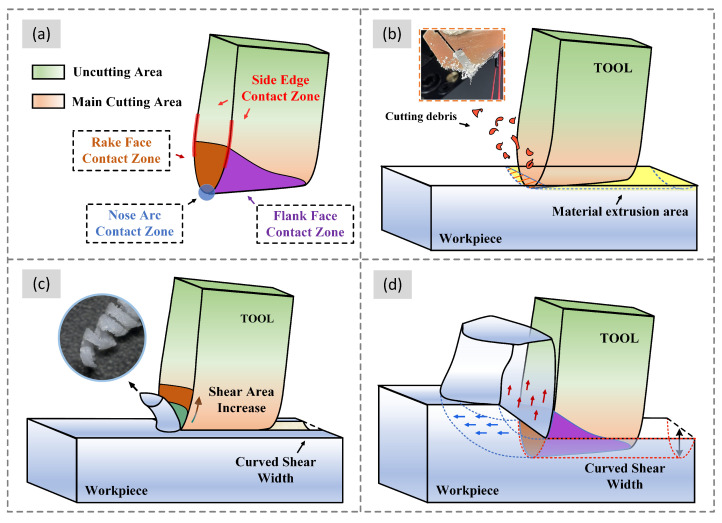
Shear behavior and chip outflow in different stages of grooving: (**a**) different contact areas of the tool at (**b**) Stage I; (**c**) Stage II; and (**d**) Stage III.

As seen in Figure 3a, the grooving insert was divided into four main contact zones: nose arc contact zone, rake face contact zone, flank contact zone, and side edge contact zone. The initial cutting process can be regarded as plowing and mainly involved contact between the tool tip arc and the workpiece (Figure 3b) [27]. Lower feed rates changed the feed depth more gradually and prolonged this stage. Therefore, $T_{L}$ was considerably higher than $T_{H}$ in Stage I. Some debris was generated in this stage because of a lack of shear. Moreover, the friction between the tool and the workpiece was dominant in this stage, thereby causing the heating of the chips and their adhesion to the tool. The rake face was in contact with the workpiece (Figure 3c); chips were generated in the contact zone between the rake face and nose radius. However, the undeformed cutting area of the curved insert changed during the cutting process, and the area of the shearing area gradually expanded [16]. The width of the chips generated because of this contact state increased with the increase in shear area. Because the increase in the feed rate accelerated the change in the undeformed cutting area, the rate of change in the cutting force increased with the increase in the feed rate in Stage II. After a certain feed depth was achieved, as illustrated in Figure 3d, the shear area of the rake face reached the maximum value. Meanwhile, the friction in the flank face also increased gradually, thereby resulting in a common contact stage of tool nose arc—rake face—flank face—workpiece. The frictional extrusion between the material and the tool changed to high—intensity shear. The feed depth, material shear, and friction increased with the increase in feed rate. Therefore, the cutting forces increase at high feed rates primarily because of friction and deformation, as observed in Stage III of cutting. Finally, with the increase in feed rate, excessive friction caused the workpiece to vibrate noticeably.

**Figure 4:**
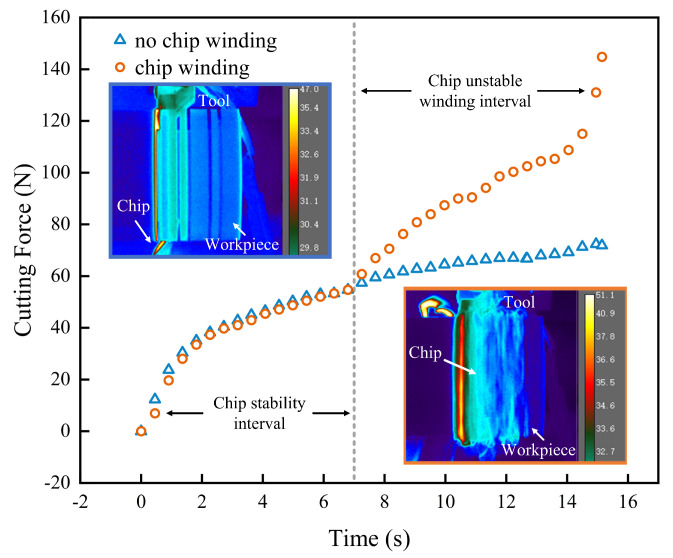
Cutting force and temperature variation during PTFE chip winding.

As shown in Figure 4, chip winding occurred at feed rates of 0.15 and 0.20 mm/rev, and the trend of the cutting force curve before winding was the same. After chip winding, excess chips accumulated between the grooves. Consequently, the vibrations in the workpiece increased, and excessive vibrations resulted in an unstable cutting force at the two aforementioned feed rates. The cutting temperature during tool feed was detected using a thermography camera, and a thermal cloud map was created. Under normal conditions, chips flowed smoothly along the contact area. The heat generated during cutting was primarily absorbed by the chips, increasing the temperature up to 35 °C in the groove. During chip curling, the dissipation of heat from the cutting area of the tool was challenging. Because of the accumulation of chips between the grooves, the maximum temperature in the groove increased to 51.1 °C. To prevent this phenomenon, feed rates of 0.15 and 0.20 mm/rev should not be used.

### 3.2. Analysis of Groove Width

**Figure 5:**
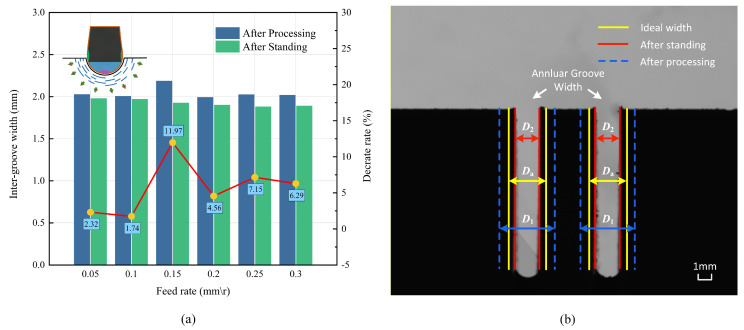
Comparison of groove width of PTFE material after 72 hours of standing: (**a**) comparison of groove width with different feeds and (**b**) time—dependent spring back.

Groove width is an important machining parameter that should be accounted for during machining. A video—measuring machine VMC432 was used to measure the width of the annular grooves from multiple angles, and averaged data were used to minimize measurement errors. The groove width decreased after cutting, and the corresponding rate of change was defined as the width decrease rate. The groove width was measured under different conditions. The groove width measured immediately after processing was denoted by $D_{1}$. As illustrated in Figure 5a, when the feed rate was 0.20, $D_{1}$ was the smallest, with a minimum value of 1.994 mm. The maximum value of $D_{1}$ was 2.028 mm at a feed rate of 0.05 mm/rev. In addition, the groove width was measured at room temperature for 72 h and denoted by $D_{2}$. When the feed rate was 0.05 mm/rev, $D_{2}$ had the maximum value. With the ideal groove width $D_{a}$ = 2, the feed rate of 0.05 mm/rev was closest to the optimal value.

As shown in Figure 5b, the groove width $D_{2}$ after 72 h of standing was smaller than $D_{1}$ after machining. The results indicated that the processed workpiece underwent material deformation, resulting in shrinkage of the inner wall. As reported in the literature, changes in groove width are caused by the time—dependent springback [28,29] of the treated surface material. Strain recovery during material processing is not linear elastic, and thereby it results in hysteresis. The width reduction rate τ at different feed rates was calculated as follows:

(1) $$\tau =\frac{D_{1}-D_{2}}{D_{1}}\times 100\%$$

As illustrated in Table 2, the changes in material width were the most notable at the feed rate of 0.15 mm/rev; the width decreased by 11.97%. The decrease at low feed rates was small, with a minimum decrease of 1.74%. The deformation due to cutting at high—feed conditions was large, thereby increasing the residual stress. The residual stress increased the degree of time—dependent springback after release. When the feed rate was 0.15 mm/rev, the curling phenomenon occurred, and the excessive vibration caused the largest deformation. Therefore, a large deformation increased the degree of the time—dependent springback. Considering the machining error, measurement error, environmental error, and other factors [30,31], by comparing the difference between the error and the change of groove width, it is determined that the time—dependent springback is the main cause of the change of groove width. The cutting quality of the external cylindrical groove is calculated by considering the error rate between the machining width and the ideal width. The error rate σ of the width at different feed rates was calculated as follows:

(2) $$\sigma =\frac{D_{a}-D_{2}}{D_{a}}\times 100\%$$

The error rate decreased with the decrease in the feed rate and had a minimum value of 0.95% at 0.05 mm/rev. According to the obtained results, the feed rate of 0.05 mm/rev was suitable for achieving the ideal groove width.

### 3.3. Chip Morphology and Surface Roughness

**Figure 6:**
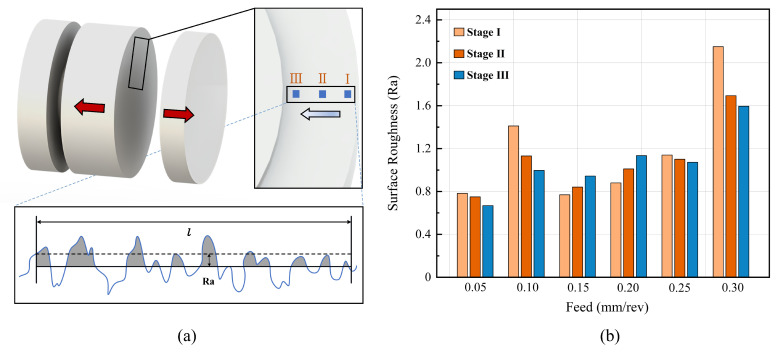
Analysis of surface roughness: (**a**) sample preparation and selection of detection area and (**b**) variation of surface roughness with respect to feed rate at various cutting stage.

Surface roughness is a key criterion for the evaluation of machining performance. The workpiece was shaped into a sheet specimen, as illustrated in Figure 6a. The groove surface roughness was calculated using the arithmetic average of the surface profile obtained using an SJ-200 roughness tester. The sampling length and cutoff length of the tester were 5 and 0.8 mm, respectively. The surface roughness was measured three times along the groove surface at different depths, corresponding to the three stages of the cutting process. The surface roughness for each group was measured four times and the mean value was calculated. Comparing the roughness at different feed rates, as illustrated in Figure 6b, the smallest and largest values of roughness at 0.05 mm/rev were obtained.

We compared the surface roughness values in different stages at the same feed rate. When the feed rate was 0.05, 0.10, 0.25, and 0.30 mm/rev, the roughness decreased gradually. Moreover, the linear cutting velocity had a notable influence on the roughness of the machined surface. During grooving, with the gradual increase in the cutting diameter, the cutting speed decreased continuously, but the angular velocity was constant. Therefore, the friction time between the tool and the side wall of the workpiece was longer in Stage III, and the roughness decreased.

When the feed rate was 0.15–0.25 mm/rev, the chip winding phenomenon occurred, and the trend of roughness change at this point was opposite to that in the normal cutting process. After the chips were entangled, the vibrations in the workpiece were aggravated. With the changes in depth, the influence of vibration on the shearing process increased, and the stability of the cutting groove decreased. Excessive chip wrapping affected the friction between the tool and the side wall of the workpiece, thereby resulting in unstable roughness.

**Figure 7:**
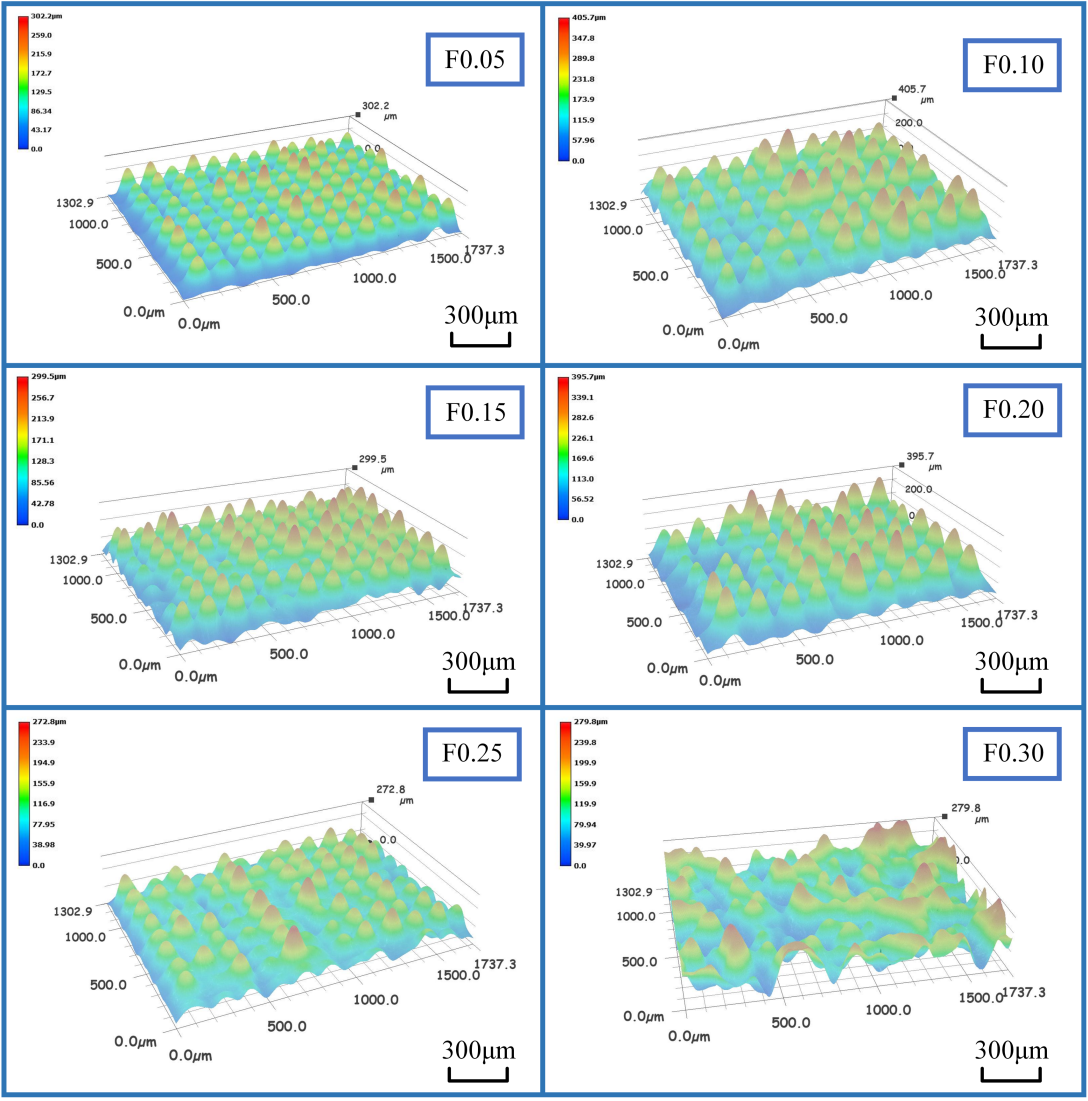
Surface waviness at different feed rates.

An optical microscope with a fixed magnification of 20 times was used to analyze the separated specimen, as displayed in Figure 6a. Surface damage and impurities were eliminated during detection. Specimen detection was performed four times for each parameter, and the 3D topography of the detected surface was output each time. Further, we investigated the micromorphology of the groove surface after cutting. As illustrated in Figure 7, an array of peaks was part of the surface structure of the PTFE grooves, as observed using an optical microscope, and the distribution of surface protrusions was the densest at a feed rate of 0.05 mm/rev. With the increase in the feed rate, the distribution of the surface protrusions became sparse, and the height of the protrusions increased because the grooving process was conducted in two directions: cut—in and cut—out. These two directions were opposite to each other, resulting in inconsistent shear directions when the material was in contact with a rotating workpiece on the spindle. The greater the feed rate, the stronger the shearing effect of the tool and material contact surface, and the smaller the density of the crest arrangement. As illustrated in Figure 6b, this densely arranged surface had higher roughness. Therefore, the surface quality was optimal at the feed rate of 0.05 mm/rev.

**Figure 8:**
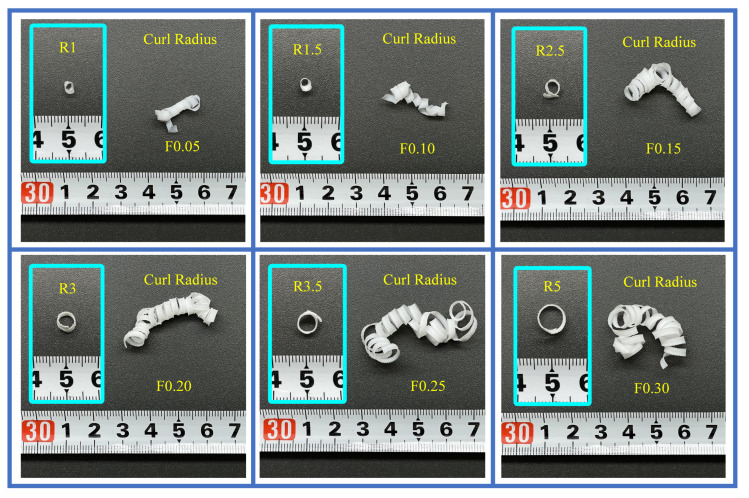
Surface waviness of groove surface in different conditions.

The feed rate affected the overall chip morphology. According to standard ISO 3685, the chip type produced by PTFE grooving was tubular type. As depicted in Figure 8, the curl radius increases with the increase of feed rate. Low feeds produce less-rigid chips due to shear, resulting in a smaller curl radius. When the feed rate is higher than 0.15 mm/rev, the curl radius increases and scales appear on the surface. As the cutting depth increases, the chip thickness increases significantly, thereby strengthening the curling resistance at the “chip–tool” interface and increasing the chips’ stiffness [32]. The chip morphology achieved under different feed rates was inconsistent, especially at 0.05 and 0.30 mm/rev. Therefore, the feed rate is the key parameter that influences chip morphology.

## 4. Conclusions

In this study, we conducted single—factor grooving experiments on PTFE using a curvilinear PCD insert. The influence of feed rate on the grooving process was assessed in terms of cutting force, groove width, chip morphology, and surface roughness. The main conclusions of the study are as follows:

- The trends in the cutting force curves at different feed rates are different because of the different contact modes used in each stage. The rapid increase in cutting force in Stage II was affected by the increase in the area of the undeformed zone.
- Tubular type chips are generated during grooving, and the chip curl radius was the largest at 5 mm when the feed rate was 0.30 mm/rev. However, the large vibrations observed at the initial stage of cutting caused chip curling and affected the stability of the cutting force curve.
- When the feed rate was 0.05 mm/rev, the groove surface roughness was optimal. The surface of the material processed at a small feed rate exhibited high density and good uniformity.
- The groove width changed over time because of the time—dependent springback. The groove width error rate increased with the increase in the feed rate. The analysis in this study revealed that 0.05 mm/rev is the most suitable feed rate for grooving.

In the analysis of cutting force in this study, the influence of temperature on the mechanical properties of materials was not considered. Future studies should focus on analyzing the dynamic mechanical properties of PTFE during the cutting process and verifying the cutting force through simulation. Furthermore, the grooving shear plane and slip zone should be characterized.

## Figures and Tables

**Table 1:** Mechanical characteristics of the workpiece.

Properties	Values
Young’s modulus [4]	0.5 GPa
Thermal conductivity [4]	0.25 W/mK
Yield strength [4]	27.4 MPa
Density [4]	2.2 kg/m^3^
Hardness [25]	55 (HS)
Poisson’s ratio [25]	0.46

**Table 2:** Groove width change at different feed rates after cutting.

Feed Rate	$D_{1}$	$D_{2}$	Decrease Rate	Error Rate
0.05	2.0084	1.981	2.32%	0.95%
0.10	2.0074	1.972	1.74%	1.40%
0.15	2.0257	1.927	11.97%	3.65%
0.20	1.9940	1.903	4.56%	4.85%
0.25	2.0268	1.882	7.15%	5.90%
0.30	2.0191	1.892	6.29%	5.80%

## Data Availability

Not applicable.

## Nomenclature

The following abbreviations are used in this manuscript:

PTFE	Polytetrafluoroethylene
PCD	Polycrystalline diamond
$T_{L}$	Duration of Stage I of low feed (s)
$T_{H}$	Duration of Stage I of high feed (s)
$K_{L}$	The cutting force slopes of Stage III at low feed rates (N/s)
$K_{H}$	The cutting force slopes of Stage III at high feed rates (N/s)
*a*	The insert length (mm)
*b*	The insert width (mm)
γ	Tool rake angle
α	Tool relief angle
*H*	The ideal groove depth (mm)
$D_{a}$	The ideal groove width (mm)
$D_{1}$	The groove width after processing (mm)
$D_{2}$	The groove width after standing (mm)
τ	The decrease rate of the groove width
σ	The error rate of the groove width

## References

[1] Dhanumalayan E., Joshi G.M. (2018). Performance Properties and Applications of Polytetrafluoroethylene (PTFE)—A Review. Adv. Compos. Hybrid. Mater..

[2] Rae P.J., Dattelbaum D.M. (2004). The Properties of Poly(Tetrafluoroethylene) (PTFE) in Compression. Polymer.

[3] Rae P.J., Brown E.N. (2005). The Properties of Poly(Tetrafluoroethylene) (PTFE) in Tension. Polymer.

[4] Cui Z., Ni J., He L., Guan L., Han L., Sun J. (2022). Investigation of Chip Formation, Cutting Force and Surface Roughness during Orthogonal Cutting of Polytetrafluoroethylene. J. Manuf. Process..

[5] Natarajan E., Kaviarasan V., Lim W.H., Tiang S.S., Parasuraman S., Elango S. (2020). Non-Dominated Sorting Modified Teaching–Learning-Based Optimization for Multi-Objective Machining of Polytetrafluoroethylene (PTFE). J. Intell. Manuf..

[6] Fetecau C., Stan F. (2012). Study of Cutting Force and Surface Roughness in the Turning of Polytetrafluoroethylene Composites with a Polycrystalline Diamond Tool. Measurement.

[7] Ni J., Han L., Wu S., Cui Z., Zeng X. (2022). Modeling of Thrust and Torque for Drilling PTFE Materials. Int. J. Adv. Manuf. Technol..

[8] Gan Y., Wang Y., Liu K., Han L., Luo Q., Liu H. (2021). A Novel and Effective Method for Cryogenic Milling of Polytetrafluoroethylene. Int. J. Adv. Manuf. Technol..

[9] Petropoulos G., Mata F., Davim J.P. (2008). Statistical Study of Surface Roughness in Turning of Peek Composites. Mater. Des..

[10] Ji S., Sun C., Zhao J., Liang F. (2015). Comparison and Analysis on Mechanical Property and Machinability about Polyetheretherketone and Carbon-Fibers Reinforced Polyetheretherketone. Materials.

[11] Jogi B.F., Tarekar M., Dhajekar R.M., Pawade R. (2016). Multi Objective Optimization Using Taguchi Grey Relational Analysis (Gra) for Cnc Turning of Poly-Ether-Ether-Ketone (Peek) Polymer. Polym. Polym. Compos..

[12] Goel B., Singh S., Sarepaka R.G.V. (2016). Precision Deterministic Machining of Polymethyl Methacrylate by Single-Point Diamond Turning. Mater. Manuf. Process..

[13] Trifunović M., Madić M., Janković P., Rodić D., Gostimirović M. (2021). Investigation of Cutting and Specific Cutting Energy in Turning of POM-C Using a PCD Tool: Analysis and Some Optimization Aspects. J. Clean. Prod..

[14] Gupta A.K., Guntuku S.C., Desu R.K., Balu A. (2015). Optimisation of Turning Parameters by Integrating Genetic Algorithm with Support Vector Regression and Artificial Neural Networks. Int. J. Adv. Manuf. Technol..

[15] Chabbi A., Yallese M.A., Nouioua M., Meddour I., Mabrouki T., Girardin F. (2017). Modeling and Optimization of Turning Process Parameters during the Cutting of Polymer (POM C) Based on RSM, ANN, and DF Methods. Int. J. Adv. Manuf. Technol..

[16] Ni J., Tong K., Meng Z., Feng K. (2022). Force Model for Complex Profile Tool in Broaching Inconel 718. Int. J. Adv. Manuf. Technol..

[17] Weng J., Zhuang K., Chen D., Guo S., Ding H. (2017). An Analytical Force Prediction Model for Turning Operation by Round Insert Considering Edge Effect. Int. J. Mech. Sci..

[18] Weng J., Zhuang K., Zhu D., Guo S., Ding H. (2018). An Analytical Model for the Prediction of Force Distribution of Round Insert Considering Edge Effect and Size Effect. Int. J. Mech. Sci..

[19] Lu L., Tang Y., Yuan D., Deng D. (2011). Groove Deformation Analysis of a Single Plough on Inner Copper Tube. J. Mater. Process. Technol..

[20] Li B., Zhang S., Yan Z., Zhang J. (2018). Effect of Edge Hone Radius on Chip Formation and Its Microstructural Characterization in Hard Milling of AISI H13 Steel. Int. J. Adv. Manuf. Technol..

[21] Xu J., Wang F., Jiang F., Chen J., Wu X., Xie H., Wang J., Lin W. (2020). Experimental Study on the Low-Feed Grooving Process with Different Grooving Tools. Int. J. Adv. Manuf. Technol..

[22] Hocheng H., Hsieh M.L. (2004). Signal Analysis of Surface Roughness in Diamond Turning of Lens Molds. Int. J. Mach. Tools Manuf..

[23] Tian F., Yin Z., Li S. (2015). Fast Tool Servo Diamond Turning of Optical Freeform Surfaces for Rear-View Mirrors. Int. J. Adv. Manuf. Technol..

[24] Zhi-jian G., Liang H., Xiao-dong R., Rui S., Xin F. (2022). The optimization of bellows convolutions in bellows pump for better stress distribution. J. Mech. Eng. Sci..

[25] DuPont Fluoroproducts (1996). Teflon PTFE Fluoropolymer Resin: Properties Handbook.

[26] Ferreira J.R., Coppini N.L., Miranda G.W.A. (1999). Machining Optimisation in Carbon ®bre Reinforced Composite Materials. J. Mater. Process. Technol..

[27] Liu K., Melkote S.N. (2006). Effect of Plastic Side Flow on Surface Roughness in Micro-Turning Process. Int. J. Mach. Tools Manuf..

[28] Tan B., Stephens L.S. (2019). Evaluation of Viscoelastic Characteristics of PTFE-Based Materials. Tribol. Int..

[29] Wang J.F., Wagoner R.H., Carden W.D., Matlock D.K., Barlat F. (2004). Creep and Anelasticity in the Springback of Aluminum. Int. J. Plast..

[30] Zawada-Tomkiewicz A., Tomkiewicz D., Pela M. (2022). Identification of a Workpiece Temperature Compensation Model for Automatic Correction of the Cutting Process. Materials.

[31] Portman V.T., Weill R.D., Shuster V.G., Rubenchik Y.L. (2003). Linear-Programming-Based Assessments of Geometrical Accuracy: Standard Presentation and Application Area. Int. J. Mach. Tools Manuf..

[32] Cui Z., Ni J., He L., Su R., Wu C., Xue F., Sun J. (2022). Assessment of Cutting Performance and Surface Quality on Turning Pure Polytetrafluoroethylene. J. Mater. Res. Technol..

